# The Impact of MiR-33a-5p Inhibition in Pro-Inflammatory Endothelial Cells

**DOI:** 10.3390/diseases11030088

**Published:** 2023-06-24

**Authors:** Kun Huang, Mark Pitman, Olanrewaju Oladosu, Jing Echesabal-Chen, Lucia Vojtech, Ikechukwu Esobi, Jessica Larsen, Hanjoong Jo, Alexis Stamatikos

**Affiliations:** 1 Department of Food, Nutrition, and Packaging Sciences, Clemson University, Clemson, SC 29634, USA; kunh@g.clemson.edu (K.H.); oolados@g.clemson.edu (O.O.); jchen11@clemson.edu (J.E.-C.); iesobi@g.clemson.edu (I.E.); 2 Department of Chemical and Biomolecular Engineering, Clemson University, Clemson, SC 29634, USA; pitman@g.clemson.edu (M.P.); larsenj@clemson.edu (J.L.); 3 Department of Obstetrics & Gynecology, University of Washington, Seattle, WA 98109, USA; luciav@uw.edu; 4 Department of Bioengineering, Clemson University, Clemson, SC 29634, USA; 5 Coulter Department of Biomedical Engineering, Georgia Institute of Technology and Emory University, Atlanta, GA 30322, USA; hjo@emory.edu

**Keywords:** endothelial activation, endothelial dysfunction, HDL, microRNA, nanoparticle, nanotherapy, reverse cholesterol transport, vascular inflammation, VCAM-1

## Abstract

Evidence suggests cholesterol accumulation in pro-inflammatory endothelial cells (EC) contributes to triggering atherogenesis and driving atherosclerosis progression. Therefore, inhibiting miR-33a-5p within inflamed endothelium may prevent and treat atherosclerosis by enhancing apoAI-mediated cholesterol efflux by upregulating ABCA1. However, it is not entirely elucidated whether inhibition of miR-33a-5p in pro-inflammatory EC is capable of increasing ABCA1-dependent cholesterol efflux. In our study, we initially transfected LPS-challenged, immortalized mouse aortic EC (iMAEC) with either pAntimiR33a5p plasmid DNA or the control plasmid, pScr. We detected significant increases in both ABCA1 protein expression and apoAI-mediated cholesterol efflux in iMAEC transfected with pAntimiR33a5p when compared to iMAEC transfected with pScr. We subsequently used polymersomes targeting inflamed endothelium to deliver either pAntimiR33a5p or pScr to cultured iMAEC and showed that the polymersomes were selective in targeting pro-inflammatory iMAEC. Moreover, when we exposed LPS-challenged iMAEC to these polymersomes, we observed a significant decrease in miR-33a-5p expression in iMAEC incubated with polymersomes containing pAntimR33a5p versus control iMAEC. We also detected non-significant increases in both ABCA1 protein and apoAI-mediated cholesterol in iMAEC exposed to polymersomes containing pAntimR33a5p when compared to control iMAEC. Based on our results, inhibiting miR-33a-5p in pro-inflammatory EC exhibits atheroprotective effects, and so precisely delivering anti-miR-33a-5p to these cells is a promising anti-atherogenic strategy.

## 1. Introduction

Atherosclerotic cardiovascular disease is a condition that kills more people worldwide than any other disease [1]. While the pathophysiology of atherosclerotic cardiovascular disease is complex and poorly understood [2], endothelial cell (EC) inflammation is recognized to contribute to both atherogenesis and atherosclerosis progression [3]. Indeed, vascular inflammation is an intriguing component of atherosclerosis, as inflammation in the EC facilitates monocyte/macrophage intimal entry, which allows macrophages to engulf intimal cholesterol [4]. This in turn may result in the accumulation of lipid-laden macrophages within the intima, leading to atherosclerosis formation [5].

Endothelial dysfunction and activation are strongly linked with EC inflammation [6,7]. Interestingly, an acknowledged precursor to endothelial dysfunction/activation is EC cholesterol accumulation, and it has even been proposed that cholesterol accumulation in EC leads to an atherogenic environment that initiates atherogenesis and exacerbates atherosclerosis [8]. In addition, EC cholesterol accumulation also stimulates vascular inflammation, resulting in EC expressing pro-inflammatory adhesion molecules commonly associated with endothelial activation [9,10,11].

Researchers have achieved pro-inflammatory endothelium-directed nanotherapy through targeting EC adhesion molecules [12,13]. A notable pro-inflammatory EC adhesion molecule that has been successfully targeted using nanoparticles is VCAM-1 [14]. Indeed, a VHPK peptide has been shown to precisely bind to VCAM-1, which permits VHPK-decorated nanoparticles to selectively bind to VCAM-1-expressing EC [15]. This accomplishment opens the possibility of constructing nanoparticles that can be internalized by pro-inflammatory EC and promote the removal of excess cholesterol from these cells, resulting in atheroprotection.

The miRNA miR-33a-5p is well-established to encourage cellular cholesterol retention in mammals. It accomplishes this by silencing ABCG1 (in rodents) as well as ABCA1 in several mammalian species, including humans [16,17,18]. While ABCG1 does not have the ability to efflux cholesterol to apoAI [19,20], ABCA1 is absolutely required for apoAI-mediated cholesterol efflux [21,22], and this inhibition of ABCA1 via miR-33a-5p expression can result in cells accumulating cholesterol. Thus, manipulating miR-33a-5p expression in cells thought to be primarily responsible for atherosclerosis formation may alter the development of atherosclerosis. Indeed, evidence supports that atheroprotection does occur when miR-33a-5p expression is ablated in macrophages, hepatocytes, and vascular smooth muscle cells [23,24,25], but data on miR-33a-5p function in EC is scant. For instance, while a recent publication shows miR-33a-5p expression may impair apoAI-mediated cholesterol efflux through ABCA1 modulation within the venous EC line HUVEC [26], it is not entirely elucidated whether miR-33a-5p plays an atherogenic role in arterial EC through the attenuation of ABCA1-dependent cholesterol efflux. Moreover, to the authors knowledge, there have been no attempts to robustly test whether miR-33a-5p expression decreases apoAI-mediated cholesterol efflux in pro-inflammatory arterial EC.

The purpose of this work was to identify miR-33a-5p expression as atherogenic in pro-inflammatory arterial EC by impairing apoAI-mediated cholesterol efflux in these cells. In this study, we also sought to construct polymer-based nanoparticles (i.e., polymersomes) capable of precisely delivering plasmids expressing anti-miR-33a-5p to pro-inflammatory EC. We challenged immortalized mouse aortic endothelial cells (iMAEC) with the pro-inflammatory stimulant LPS to induce VCAM-1 expression. When anti-miR-33a-5p was delivered to these cultured iMAEC via plasmid transfections, we observed a significant increase in apoAI-mediated cholesterol efflux. Furthermore, when we used VCAM-1 binding polymersomes to deliver anti-miR-33a-5p-expressing plasmid DNA to LPS-challenged iMAEC, we observed a significant decrease in miR-33a-5p expression along with statistical trends for ABCA1 protein and apoAI-mediated cholesterol efflux. These results imply that miR-33a-5p expression in pro-inflammatory endothelium is atherogenic, and delivering anti-miR-33a-5p to these cells using nanoparticles may be a promising strategy for treating atherosclerosis.

## 2. Materials and Methods

### 2.1. Maintenance and Treatment of iMAEC

The iMAEC [27] were provided by the Jo Laboratory and were cultured as described [28]. We also purchased primary mouse aortic endothelial cells (pMAEC) from Cell Biologics (Chicago, IL, USA) and cultured/maintained these cells as described [28] so that they could be utilized as a positive control to confirm miR-33a-5p expression and function were present within iMAEC. For all in vitro experiments involving iMAEC, we allowed the cells to expand to 70–80% confluency before initiating treatments. We stimulated pro-inflammatory conditions within iMAEC by challenging the cells with LPS (10 ng/mL; Sigma-Aldrich, St. Louis, MO, USA) to compare with vehicle-control-treated iMAEC. Twenty-four hours after iMAEC were exposed to LPS/vehicle, we washed the cells with PBS and then either transfected the iMAEC with plasmid DNA or incubated the cells with polymersomes. For the transfections, we used two different plasmids (System Biosciences, Palo Alto, CA, USA), both containing an expression cassette that included an H1 promoter and a 5T termination signal [25], and transfected these plasmids into iMAEC by using jetOPTIMUS (Polyplus, New York, NY, USA), as previously described [29]. The control plasmid used expresses a non-targeting scrambled anti-miR (pScr), while the other plasmid expresses ant-miR-33a-5p (pAntimiR33a5p) [25]. Post-transfection, we harvested the iMAEC to collect total RNA after 24 h or collected lysates 48 h after transfections for measuring protein expression. For polymersome incubation, we exposed iMAEC to the nanoparticles (1 polymersome for every iMAEC) for 1 h, washed cells with PBS, replenished cells with standard growth medium, and then collected DNA/RNA, or protein from cells after 24 h or 48 h, respectively.

### 2.2. Polymersome Construction and Characterization

For materials, we used poly(ethylene glycol)_1000_-*block*-poly(lactic acid)_5000_ (PEGPLA; PolySciences, Inc., Warrington, PA, USA) and maleimide-functionalized poly(ethylene glycol)_1000_-*block*-poly(lactic acid)_5000_ (mal-PEGPLA; Broadpharm, San Diego, CA, USA) for polymersome formation. N,N-dimethylformamide (DMF; VWR, Radnor, PA, USA) and D-mannitol (Fisher Chemical, Fair Lawn, NJ, USA) were also used during polymersome synthesis. For peptide-decorated polymersomes, VHPKQHRGGSKGC (VHPK) peptide was purchased from GenScript (Piscataway, NJ, USA) [15].

We formed the polymersomes by solvent injection following our previously reported method [30] with minor modifications. For undecorated polymersomes, PEGPLA was dissolved in DMF for a final concentration of 2 mM. Dissolved PEGPLA was injected by syringe pump into a 2 *w*/*v*% mannitol solution as a lyoprotectant at a rate of 5 μL/min through a 27G 0.5 mL syringe (BD, Franklin Lakes, NJ, USA). Polymersomes were dialyzed against 2 *w*/*v*% mannitol for 16 h at 4 °C in 300 kD Float-a-Lyzers (Repligen) to remove DMF. After dialysis, the polymersomes were slowly frozen at −20 °C before being moved to −80 °C. After freezing, polymersomes were lyophilized and stored at room temperature in a vacuum desiccator.

VHPK-decorated polymersomes were prepared following the same procedure, with additional steps for VHPK conjugation. VHPK was conjugated to mal-PEGPLA by the maleimide-thiol reaction with the cysteine at the end of the VHPK peptide. Prior to solvent injection, 2 mM mal-PEGPLA in DMF was combined with 1 mg/mL VHPK peptide in DMF at a 1:10 molar ratio. The mixture was stirred at room temperature for an hour. After one hour, the VHPK-conjugated PEGPLA (VHPK-PEGPLA) was combined with 2 mM PEGPLA to obtain a 50:50 ratio of VHPK-PEGPLA to PEGPLA. Polymersomes were generated and stored under the conditions listed above for undecorated polymersomes, with the dialysis step used to remove DMF and unreacted VHPK, and then post-attachment of the VHPK peptide being assessed via spectrophotometry [31].

Both sets of lyophilized polymersomes were loaded with either pScr or pAntimiR33a5p to create the four following types of polymersomes: (1) PLMRpScr; (2) PLMRpA5p; (3) VHPK-PLMRpScr; and (4) VHPK-PLMRpA5p. Briefly, 20 μL of 1 mg/mL circular plasmid DNA was added to 10 mg of lyophilized polymersomes and gently vortexed. Polymersomes were then reconstituted in 980 μL of water to obtain a final concentration of approximately 10 mg/mL. Loaded polymersomes were dialyzed against water overnight in 1000 kD Float-a-Lyzers (Repligen, Rancho Dominguez, CA, USA) to remove the unencapsulated plasmid. To determine encapsulation efficiency, a 100-microliter sample of dialyzed polymersomes was aliquoted, dissolved in 300 μL dimethyl sulfoxide (DMSO; Sigma-Aldrich, St. Louis, MO, USA), and added to a clear, UV-transparent 96-well plate. Absorbances were measured at 260 nm and 280 nm on a Biotek Synergy H1M microplate reader (Agilent, Santa Clara, CA, USA) and compared to a calibration curve for the appropriate plasmid. Encapsulation efficiency was calculated as:

$$EE\left(\%\right)=\frac{Final\;mass\;in\;polymersomes}{Initial\;mass\;added}$$

where the initial and final masses are the amount of plasmid originally added to the polymersomes and the amount encapsulated, respectively. The remaining loaded polymers were concentrated by centrifugation in Amicon 100 kD centrifugal filters (Sigma-Aldrich, St. Louis, MO, USA) and either used immediately or stored at 4 °C. Particle size and number of our polymersome preparations were assessed as previously described [25,32] by using nanoparticle tracking analysis via the NanoSight NS300 instrument (Malvern Instruments, Malvern, UK), and polymersome physical characteristics (e.g., morphology) were analyzed with transmission electron microscopy [25,32].

### 2.3. End-Point RT-PCR and RTq-PCR

To assess miR-33a-5p expression within iMAEC, we first extracted total RNA from iMAEC and pMAEC positive control cells using a Zymo Research Direct-zol total RNA column-purification kit (Irvine, CA, USA). We used this isolated total RNA to convert small RNA species into cDNA by using a QuantaBio qScript™ miRNA cDNA Synthesis kit (Beverly, MA, USA). We subsequently used this cDNA to conduct end-point PCR for the amplification of miR-33a-5p. The PCR products either remained non-digested or attempted to be digested via the restriction enzymes *Bsr*DI and *Tsp*RI (New England Biolabs, Ipswich, MA, USA) [33]. We analyzed all the amplicons and digested fragments with TBE-agarose gel (3.5%) electrophoresis using a GelDoc machine (Analytik Jena US, Upland, CA, USA). To quantify the small RNA miR-33a-5p, anti-Scr, anti-miR-33a-5p, and U6 reference genes via qPCR, we used the same RNA column-purification and cDNA synthesis kits as described above, along with a forward primer specific for these respective genes in our qPCR reactions. To quantify plasmid DNA and GADPH gDNA in transfected iMAEC and iMAEC exposed to the polymersomes, we extracted DNA with Lucigen DNA QuickExtract (Middleton, WI, USA) to use for our qPCR reactions. For all our PCR reactions described above, we used a QuantaBio PerfeCTa SYBR Green FastMix kit, with our reactions being completed by utilizing a qTOWER³ G touch qPCR instrument (Analytik Jena US) and data analyses using the ΔΔ^CT^ method [34].

To assess the silencing capabilities of miR-33a-5p for ABCA1 within iMAEC, we used purified total RNA from both iMAEC and pMAEC as a positive control for end-point RT-PCR reactions that involved the use of a QuantaBio qScript XLT 1-Step RT-PCR kit. For these reactions, the sequence that was amplified included the ABCA1 3′UTR, which exhibits miR-33a-5p binding sites [33]. These PCR products were then used to confirm the conserved miR-33a-5p binding sites are present via sequencing (Eton Bioscience, San Diego, CA, USA). The primers used for all of our PCR reactions are depicted in Table 1.

### 2.4. Western Blotting

We collected iMAEC lysates and quantified proteins within these lysates as previously described [28]. Proteins within lysates were separated by SDS-PAGE, and we transferred these proteins onto PVDF membranes (Merck Millipore Ltd., Burlington, MA, USA). After incubating the membranes in blocking buffer, we probed the blots using the following primary antibodies: ABCA1 (1:1000 dilution, sc-58,219; Santa Cruz Biotechnology, Dallas, TX, USA); GAPDH (1:1000 dilution, sc-365,062; Santa Cruz Biotechnology); and VCAM-1 (1:1000 dilution, sc-13,160; Santa Cruz Biotechnology). After probing the blots with the respective primary antibodies, we incubated the blots with HRP-conjugated goat anti-mouse IgG secondary antibodies (1:15,000 dilution, AP181P; Sigma-Aldrich, St. Louis, MO, USA). We subsequently exposed our blots to ECL substrate (Immobilon ECL Ultra Western HRP Substrate; MilliporeSigma, Billerica, MA, USA) and used a ChemiDoc instrument (Analytik Jena US) for imaging analysis. NIH ImageJ software (version 1.53a)was utilized for immunoblot densitometry.

### 2.5. Cholesterol Efflux

To introduce plasmids to cells, vehicle-/LPS-treated iMAEC were either transfected with plasmid DNA or exposed to polymersomes as described earlier. After respective treatments, we washed iMAEC with PBS and then cholesterol-loaded cells using [^3^H]cholesterol (1 μCi/mL; PerkinElmer, Waltham, MA, USA) diluted in efflux medium [35]. Twenty-four hours after [^3^H]cholesterol loading, we again washed the cells with PBS and exposed them to either apoAI (5 μg/mL; Academy Bio-Medical Company, Houston, TX, USA) or vehicle only diluted in efflux medium. After treating iMAEC with either apoAI or vehicle only for twenty-four hours, we harvested both medium and cells to measure apoAI-mediated cholesterol efflux, as previously described [28,35]. The liquid scintillation counter we used to count [^3^H] was a PerkinElmer Tri-Carb 4910TR.

### 2.6. Statistical Analyses

For statistical analysis, we used SigmaPlot software (Systat Software Inc. v14.0, San Jose, CA, USA). We initially performed a Shapiro-Wilk normality test and a Brown-Forsythe equal variance test. When both tests indicated normality and equal variance, we subsequently performed a two-tailed Student’s *t*-test. Alternatively, we performed a Mann-Whitney rank-sum test when the assumption of normality was not met and a two-tailed Welch’s t-test when equal variance was violated. We set statistical significance at *p* < 0.05.

## 3. Results

### 3.1. iMAEC Express MiR-33a-5p with the Capacity to Silence ABCA1 and Robustly Express VCAM-1 Protein When Challenged with LPS

**Figure 1:**
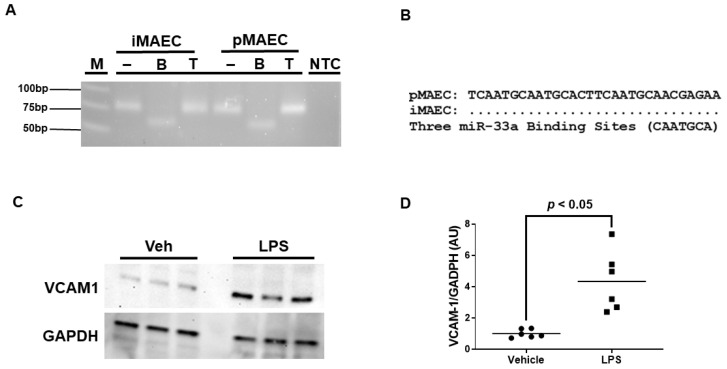
iMAEC expresses functional miR-33a-5p and exhibits pro-inflammatory properties in response to LPS exposure. (**A**) End-point RT-PCR and restriction digestion of amplicons assessed by agarose gel electrophoresis to detect miR-33a-5p within iMAEC and pMAEC positive controls. MiR-33a-5p cDNA contains one BsrDI restriction site but lacks TspRI restriction sites. M, DNA ladder; NTC, non-template control PCR reaction; minus (−), undigested amplicons; B, BsrDI-digested amplicons; T, TspRI-digested amplicons. (**B**) Three highly conserved miR-33a-5p binding sites are recognized within the 3′UTR of the ABCA1 gene for iMAEC and pMAEC positive controls. (**C**,**D**) Representative immunoblot (**C**) and densitometry (**D**) of vehicle-treated and LPS-challenged iMAEC for the quantification of VCAM-1 protein with GAPDH loading control. (**D**) Data points indicate two independent treatments with three biological replicates per respective treatment. Bars are group means, and Welch’s t-test was used to perform statistical analysis. AU, arbitrary units.

We previously reported that iMAEC are capable of participating in ABCA1-dependent cholesterol efflux [28], but it is unclear whether iMAEC express the miRNA miR-33a-5p. Using end-point RT-PCR and restriction digestion, we confirmed that iMAEC does indeed express miR-33a-5p (Figure 1A). However, for miR-33a-5p to downregulate ABCA1 protein expression, conserved miR-33a-5p binding sites within the 3′UTR of the ABCA1 gene would have to be present [16,17]. Thus, sequencing analyses were performed, which did uncover three distinct highly conserved miR-33a-5p binding sites in the 3′UTR of ABCA1 [16,17,33] for iMAEC, which demonstrates that miR-33a-5p retains the functional ability to silence ABCA1 within this immortalized cell line (Figure 1B).

Since we aimed to introduce anti-miR-33a-5p into pro-inflammatory iMAEC, which largely express VCAM-1, we first needed to expose these cells to a pro-inflammatory stimulus able to induce high levels of VCAM-1 protein. We chose to incubate iMAEC with LPS, as this molecule is well-recognized to stimulate inflammation and trigger VCAM-1 expression in endothelium [36]. As expected, when we incubated iMAEC with LPS, this resulted in a significant increase in VCAM-1 protein expression when compared to iMAEC treated with vehicle only (Figure 1C,D). Therefore, this suggests that challenging iMAEC with LPS robustly induces pro-inflammatory responses in these immortalized cells.

### 3.2. Pro-Inflammatory iMAEC Transfected with pAntimiR33a5p Exhibits Enhanced ABCA1-Dependent Cholesterol Efflux

**Figure 2:**
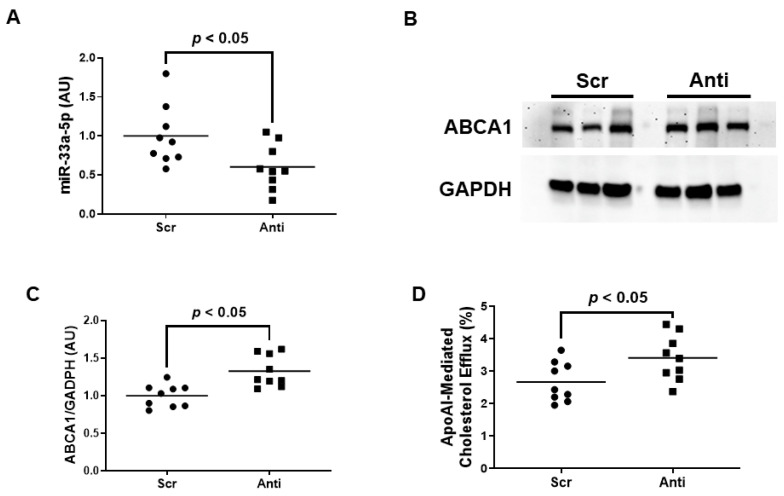
Transfecting inflamed iMAEC with pAntimiR33a5p enhances apoAI-mediated cholesterol efflux. LPS-challenged iMAEC were transfected with either pScr (Scr) or pAntimiR33a5p (Anti). (**A**) MiR-33a-5p expression measured in transfected pro-inflammatory iMAEC via RT-qPCR. (**B**,**C**) Representative immunoblot (**B**) and densitometry (**C**) of transfected pro-inflammatory iMAEC for the quantification of ABCA1 protein with GAPDH loading control. (**D**) Percent apoAI-mediated cholesterol efflux measured in transfected pro-inflammatory iMAEC. (**A**,**C**,**D**) Data points indicate three independent treatments with three biological replicates per respective treatment. Bars are group means, and a Student’s *t*-test was used to perform statistical analysis. (**A**,**C**) AU, arbitrary units.

Very little is known about the impact of miR-33a-5p expression on ABCA1 expression and apoAI-mediated cholesterol efflux within inflamed endothelium. We therefore wanted to initially assess whether inhibiting miR-33a-5p by using plasmid DNA transfection technology is successful in increasing ABCA1-dependent cholesterol efflux in pro-inflammatory EC before attempting to deliver anti-miR-33a-5p with nanoparticles. In these initial sets of experiments, we transfected LPS-challenged iMAEC with either pAntimiR33a5p or the control vector pScr. We observed a significant decrease in miR-33a-5p expression in iMAEC transfected with pAntimiR33a5p when compared to iMAEC transfected with pScr (Figure 2A). This decreased level of miR-33a-5p observed in pAntimiR33a5p-transfected iMAEC was inversely correlated with ABCA1 protein expression in these cells, as ABCA1 protein was significantly increased in this group when compared to the control group (Figure 2B,C). We also showed a significant increase in apoAI-mediated cholesterol efflux in iMAEC transfected with pAntimiR33a5p when compared to the control iMAEC (Figure 2D). Taken together, these results indicate that inhibiting miR-33a-5p expression in pro-inflammatory endothelium upregulates ABCA1 protein, resulting in enhanced apoAI-mediated cholesterol efflux.

### 3.3. VHPK-Decorated Polymersomes Are Capable of Selectively Delivering Plasmids to Pro-Inflammatory Endothelium

**Figure 3:**
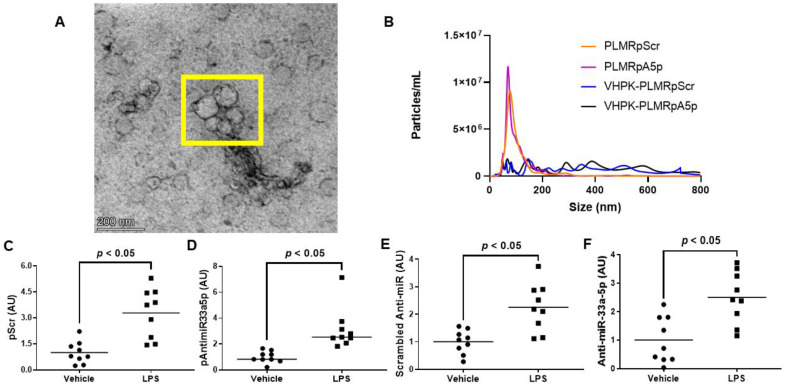
Characterization and cultured endothelial cell internalization efficiency of polymersome preparations. (**A**) Transmission electron micrograph of VHPK-decorated polymersomes. The polymersomes enclosed within the yellow box imply the aggregation of particles. (**B**) Particle number and size distribution of polymersome preparations determined by nanoparticle tracking analysis. (**C**–**F**) Plasmid DNA content (**C**,**D**) and transgene expression levels (**E**,**F**) were measured by qPCR or RT-qPCR, respectively, in vehicle-treated or LPS-challenged iMAEC exposed to either VHPK-PLMRpScr (**C**,**E**) or VHPK-PLMRpA5p (**D**,**F**). Data points indicate three independent treatments with three biological replicates per respective treatment. AU, arbitrary units. (**C**) Bars are group means, and Welch’s t-test was used to perform statistical analysis. (**D**) Bars are group medians, and a Mann-Whitney rank-sum test was used to perform statistical analysis. (**E**,**F**) Bars are group means, and a Student’s *t*-test was used to perform statistical analysis.

Since atherogenesis and atherosclerosis progression occur in areas with high levels of vascular inflammation [37,38,39,40,41], delivering atheroprotective transgenes precisely to these sites of the vessel wall would likely demonstrate atheroprotection more so than the delivery of transgenes throughout the entire arterial tree. Hence, we constructed polymersomes that have the capacity to specifically target inflamed EC by decorating these nanoparticles with a VHPK peptide that binds to the inflammatory EC adhesion molecule VCAM-1 with high affinity [14,15]. These VHPK-decorated polymersomes also either contained pScr or pAntimiR33a5p plasmid DNA, resulting in the formation of VHPK-PLMRpScr and VHPK-PLMRpA5p, respectively. We assessed plasmid encapsulation efficiencies within these two preparations and their undecorated counterparts, PLMRpScr and PLMRpA5p, and observed similar levels of plasmid DNA within intact polymersomes among all four preparations (Table 2). When we imaged the potentially therapeutic VHPK-decorated polymersomes via transmission electron microscopy, the polymersomes exhibited distinct physical characteristics that are found within intact polymersomes [42,43]. Interestingly, we also discovered potential polymersome aggregation within these VHPK-decorated polymersome preparations (Figure 3A). Thus, we further characterized our polymersome preparations by measuring particle size and number using nanoparticle tracking analysis by NanoSight [25,32] and noticed both PLMRpScr and PLMRpA5p being essentially monodisperse, but VHPK-PLMRpScr and VHPK-PLMRpA5p demonstrating a polydisperse pattern instead (Figure 3B).

To test the delivery specificity of the VHPK-decorated polymersomes within inflamed endothelium, we incubated vehicle-treated iMAEC versus LPS-challenged iMAEC with either VHPK-PLMRpScr or VHPK-PLMRpA5p and measured plasmid levels in these cells. We observed significant increases in plasmid DNA content within LPS-challenged iMAEC when compared to the control groups (Figure 3C,D). We also detected significant increases in respective shRNA transgene levels within the LPS-challenged iMAEC versus shRNA transgene levels measured in control cells (Figure 3E,F). Our results imply that selective internalization of VHPK-coated polymersomes precisely occurs within inflamed EC.

### 3.4. Impact of Polymersome-Mediated Anti-miR-33a-5p Delivery on Pro-Inflammatory EC

**Figure 4:**
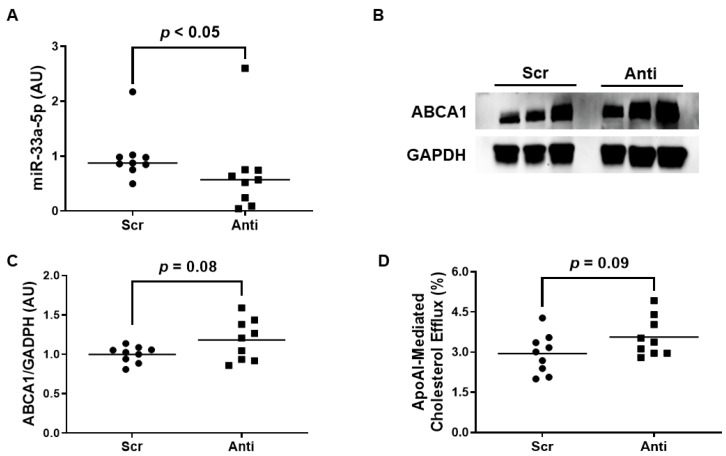
Exposing pro-inflammatory iMAEC to VHPK-PLMRpA5p moderately improves ABCA1-dependent cholesterol efflux. LPS-challenged iMAEC were incubated with either VHPK-PLMRpScr (Scr) or VHPK-PLMRpA5p (Anti). (**A**) MiR-33a-5p expression measured within inflamed iMAEC via RT-qPCR. (**B**,**C**) Representative immunoblot (**B**) and densitometry (**C**) of inflamed iMAEC for ABCA1 protein quantification with GAPDH loading control. (**D**) Percent apoAI-mediated cholesterol efflux measured within inflamed iMAEC. (**A**,**C**,**D**) Data points indicate three independent treatments with three biological replicates per respective treatment. (**A**) Bars are group medians, and a Mann-Whitney rank-sum test was used to perform statistical analysis. (**C**) Bars are group means, and Welch’s t-test was used to perform statistical analysis. (**D**) Bars are group means, and a Student’s *t*-test was used to perform statistical analysis. (**A**,**C**) AU, arbitrary units.

We exposed the LPS-challenged to either VHPK-PLMRpScr or VHPK-PLMRpA5p to assess whether this form of therapy exhibits any atheroprotective properties. When we measured miR-33a-5p levels in these treated cells, we observed a significant decrease in miR-33a-5p expression in iMAEC incubated with VHPK-PLMRpA5p when compared to iMAEC exposed to VHPK-PLMRpScr (Figure 4A). However, while anti-miR-33a-5p delivery in VHPK-PLMRpA5p-treated iMAEC did increase ABCA1 protein as well, this increase in expression was considered non-significant (Figure 4B,C). Furthermore, we also detected a trend in apoAI-mediated cholesterol efflux within iMAEC incubated with VHPK-PLMRpA5p when compared to iMAEC exposed to VHPK-PLMRpScr (Figure 4D), and so we infer that our form of polymersome-mediated anti-miR-33a-5p delivery to pro-inflammatory endothelium modestly improves ABCA1-dependent cholesterol efflux.

## 4. Discussion

In our study, we wanted to test whether inhibiting miR-33a-5p in pro-inflammatory cultured EC has the potential to increase ABCA1-dependent cholesterol efflux and if we are successful in utilizing polymersome-based strategies to selectively deliver anti-miR-33a-5p-expressing plasmids to inflamed EC. We report that transfecting LPS-challenged iMAEC with pAntimiR33a5p significantly increases both ABCA1 protein expression and apoAI-mediated cholesterol efflux, with this outcome likely being the result of a significant downregulation of miR-33a-5p expression, which was also observed in these cells. Moreover, when we incubated LPS-challenged iMAEC with VHPK-PLMRpA5p, which are polymersomes containing pAntimiR33a5p that are designed to target pro-inflammatory endothelium, we showed a significant increase in both pAntimiR33a5p levels and anti-miR-33a-5p expression when compared to iMAEC exposed to VHPK-PLMRpA5p (but pre-treated with vehicle only). In addition, we also observed a significant decrease in miR-33a-5p expression along with moderately enhanced ABCA1-dependent cholesterol efflux within the LPS-challenged iMAEC exposed to VHPK-PLMRpA5p versus the LPS-challenged iMAEC incubated with the control polymersome, VHPK-PLMRpScr.

Poor cholesterol efflux capacity is well recognized to spur atherosclerosis-related cardiovascular events [44]. Indeed, sufficient removal of excess cholesterol from arteries via cholesterol efflux is touted as being atheroprotective, while inadequate arterial cholesterol efflux may likely increase atherosclerosis development [45]. However, the impact of EC cholesterol efflux upon atherogenesis and atherosclerosis progression appears to be mainly ignored in comparison to cholesterol efflux regulation within macrophages and hepatocytes [46,47]. For instance, while there has been some published data illustrating endothelial ABCA1 expression to be atheroprotective [9,35], scant data has been published on the impact of miR-33a-5p regulating ABCA1 expression in EC when compared to the vast amount of published literature on miR-33a-5p function within hepatocytes and macrophages [26,48,49,50]. To our knowledge, no direct, rigorous testing involving miR-33a-5p inhibition within pro-inflammatory arterial EC has been performed, which is critical in the context of atherosclerosis, as this disease predominantly forms in areas exhibiting vascular inflammation. In this study, inhibiting miR-33a-5p in pro-inflammatory cultured EC does demonstrate the ability to increase apoAI-mediated cholesterol efflux, which is likely the result of ABCA1 upregulation, and thus inhibition of miR-33a-5p within inflamed endothelium may be atheroprotective by promoting the removal of excess cholesterol from inflamed atherosclerotic lesions.

A therapeutic approach we attempted in our in vitro study was to precisely deliver pAntimiR33a5p to pro-inflammatory cultured EC by utilizing polymersomes. While we achieved selective delivery of VHPK-PLMRpA5p to inflamed iMAEC, we only observed moderate increases in ABCA1-dependent cholesterol efflux. A possible reason why polymersome-mediated delivery of pAntimR33a5p to LPS-challenged iMAEC was less effective at increasing ABCA1-dependent cholesterol than plasmid transfection may be due to less plasmid DNA being delivered to inflamed iMAEC when using nanoparticles when compared to plasmid DNA transfections. Therefore, increasing the number of VHPK-PLMRpA5p particles inflamed EC are exposed to and/or increasing pAntimiR33a5p encapsulation efficiency as well as overall plasmid mass may result in augmenting ABCA1-dependent cholesterol efflux within pro-inflammatory EC incubated with these polymersomes. The original plasmid mass used in our studies was based on our prior established research, which involved successful enzymatic loading of polymersomes to be used as a form of enzyme replacement therapy [30]. However, since our delivery system uses plasmid DNA, a higher mass of plasmid may be needed for effective atheroprotection, and thus utilizing higher amounts of plasmid for polymersome loading may be warranted. A major benefit associated with polymersomes is their ability to provide variable dosing; hence, more alternative and potentially optimal doses of VHPK-PLMRpA5p could be explored to increase polymersome-mediated cellular plasmid DNA internalization. Another possibility is that we only observed modestly enhanced ABCA1-dependent cholesterol efflux in LPS-challenged iMAEC exposed to VHPK-PLMRpA5p, which may be due to polymersome aggregation potentially interfering with nanoparticle internalization within the cells. The characterization of the polymersomes did show some aggregation being present in our transmission electron micrographs and nanoparticle tracking analysis data, with the latter finding being detected due to the polydisperse nature of the polymersome preparations. Interestingly, though, only the VHPK-decorated polymersomes showed these types of polydisperse patterns, which may possibly prevent these aggregates from becoming successfully internalized. This consequence may be reflected in the miR-33a-5p expression levels within LPS-challenged iMAEC exposed to VHPK-PLMRpA5p. While we did observe a significant decrease in these treated iMAEC when compared to the control group, the data was more variable than in LPS-challenged iMAEC transfected with pAntimiR33a5p, and so the lack of aggregates becoming efficiently internalized by the cultured cells may have caused such high variability in miR-33a-5p expression levels. Thus, strategies to eliminate aggregation in the VHPK-PLMRpA5p preparations are likely warranted. A possible strategy to possibly accomplish this is by increasing the ratio of VHPK-PEGPLA to PEGPLA, which may provide more uniform surface charges on polymersomes that may result in decreased aggregation.

As we envision eventually utilizing polymersome-mediated approaches to precisely deliver anti-miR-33a-5p to pro-inflammatory EC in vivo to protect against atherosclerosis, a major limitation to our study is not testing whether this polymersome-based strategy alters EC physiology. Since we are experienced with assessing the potential toxicity of proposed atheroprotective therapeutics in cultured EC [35], we acknowledge that the most optimal method to conduct these types of experiments is with primary EC instead of utilizing immortalized cell lines like we did in our study [51]. Furthermore, a definite challenge to analyzing toxicity in our cell culture model is the use of LPS to induce endothelial inflammation since LPS is known to be directly toxic to cultured EC [52,53] and can trigger EC dysfunction [54]. Therefore, an alternative approach to testing whether polymersome-based delivery of anti-miR-33a-5p is directly toxic to pro-inflammatory EC is to assess crucial physiological EC functions within cultured primary arterial EC exposed to PLMRpA5p, so that we may indirectly deduce the safety profile of VHPK-PLMRpA5p. Another important item to mention is that LPS appears to increase miR-33a-5p expression within cultured cells [55,56], and so alternative approaches to induce VCAM-1 expression in vitro that are known not to influence miR-33a-5p expression should be considered when optimizing VHPK-PLMRpA5p delivery within cultured EC.

If our delivery strategy is further optimized to promote robust increases in ABCA1-dependent cholesterol efflux within pro-inflammatory EC, it is possible that the enhanced effect of apoAI-mediated cholesterol efflux in these targeted cells may cause VCAM-1 downregulation. Indeed, we and others have shown that ABCA1 expression is anti-inflammatory via decreasing cellular lipid raft content through its participation in apoAI-mediated cholesterol efflux [35,57,58,59], and this effect is capable of decreasing VCAM-1 expression as well as the expression of other pro-inflammatory NF-κB target genes. An intriguing predicament is the possibility that over time the potential atheroprotective impact of VHPK-PLMRpA5p delivery may be diminished if chronic VCAM-1 suppression occurs within EC residing in atheroprone arteries and atherosclerotic lesions, as this may prevent entry of anti-miR-33a-5p into these cells via impeding efficient internalization of VHPK-PLMRpA5p. Hence, pending VHPK-PLMRpA5p optimization, time-course studies involving atherogenic animal models should be utilized to directly test whether VHPK-PLMRpA5p delivery can treat atherosclerosis, both acutely and over the long term.

In conclusion, inhibition of miR-33a-5p within inflamed endothelium appears to demonstrate atheroprotective qualities via enhancing apoAI-mediated cholesterol efflux through ABCA1 upregulation in pro-inflammatory EC. Moreover, nanoparticle-mediated delivery of anti-miR-33a-5p may be a powerful atheroprotective tool for treating atherosclerosis. Hence, future studies should be conducted that directly test whether precisely delivering anti-miR-33a-5p to pro-inflammatory EC in atherogenic animal models may effectively treat atherosclerosis. If selective delivery of VHPK-PLMRpA5p to pro-inflammatory EC can be accomplished through systemic delivery and result in safely treating atherosclerosis, then this may indeed be an advancement over attempting to manipulate arterial wall gene expression through viral vector-based systemic administration [60].

## Figures and Tables

**Table 1:** Primer pairs.

Target	Template	Sequence (5′-3′)
MiR-33a-5p	cDNA	forward: CGCGTGCATTGTAGTTGCATTGC
Anti-miR-33a-5p	cDNA	forward: TGCAATGCAACTACAATGCAC
Scrambled Anti-miR	cDNA	forward: TAAGGTTAAGTCGCCCTCGC
U6	cDNA	forward: TGGCCCCTGCGCAAGGATG
Global Small RNA	cDNA	reverse: GCATAGACCTGAATGGCGGTA
ABCA1 3′ UTR	cDNA	forward: AAGAGCGAGGTCTTCCTTTG
		reverse: TGGCTTAATGGACGAGGATG
GAPDH	gDNA	forward: GTGTCACTACCGAAGAAC
		reverse: AGGACTCAGGGAATACAG
pAntimiR33a5p/pScr	pDNA	forward: GCTTAACTATGCGGCATCAGAG
		reverse: TAATCGCCTTGCAGCACATC

**Table 2:** Percent polymersome encapsulation efficiency (EE).

Polymersome Preparations	Preparation 1 EE	Preparation 2 EE
PLMRpScr	9%	14%
VHPK-PLMRpScr	10%	15%
PLMRpA5p	14%	15%
VHPK-PLMRpA5p	7%	12%

## Data Availability

All represented data in our study is enclosed within the manuscript.
